# Mycobacterial escape from macrophage phagosomes to the cytoplasm represents an alternate adaptation mechanism

**DOI:** 10.1038/srep23089

**Published:** 2016-03-16

**Authors:** Shilpa V. Jamwal, Parul Mehrotra, Archana Singh, Zaved Siddiqui, Atanu Basu, Kanury V.S. Rao

**Affiliations:** 1 Immunology Group International Centre for Genetic Engineering and Biotechnology, Aruna Asaf Ali Marg, New Delhi, 110067, India; 2 Systems Biology Group Institute of Genomics and Integrative Biology, Mall Road, New Delhi, 110007, India; 3 National Institute of Virology Dr. Babasaheb Ambedkar Road, Pune, 411001, India

## Abstract

Survival of *Mycobacterium tuberculosis* (Mtb) within the host macrophage is mediated through pathogen-dependent inhibition of phagosome-lysosome fusion, which enables bacteria to persist within the immature phagosomal compartment. By employing ultrastructural examination of different field isolates supported by biochemical analysis, we found that some of the Mtb strains were in fact poorly adapted for subsistence within endocytic vesicles of infected macrophages. Instead, through a mechanism involving activation of host cytosolic phospholipase A_2_, these bacteria rapidly escaped from phagosomes, and established residence in the cytoplasm of the host cell. Interestingly, by facilitating an enhanced suppression of host cellular autophagy, this translocation served as an alternate virulence acquisition mechanism. Thus, our studies reveal plasticity in the adaptation strategies employed by Mtb, for survival in the host macrophage.

Subsequent to its uptake, inhibition of phagosome-lysosome fusion is central to the survival of *Mycobacterium tuberculosis* (Mtb) within human macrophages. This is achieved by pathogen-mediated manipulation of host signaling pathways, which ensures that the bacteria remain in early endosome^1^,^2^,^3^,^4^,^5^. Evidence has, however, emerged over the years to suggest that mycobacteria may eventually escape from phagosomes by translocating to the cytosol. Initial observations to this effect^6^,^7^,^8^ were more recently corroborated, with the dynamics of the process also being characterized. These latter studies have additionally revealed that cytosolic translocation of Mtb occurs in the late stage of infection, and that it might reflect a virulence mechanism of the pathogen^5^,^9^,^10^,^11^.

In spite of growing support for the cytosolic translocation of Mtb, unanswered questions pertaining to its biological relevance remain. Barring one exception^9^, studies of this phenomenon employed either laboratory strains of Mtb, or the fish pathogen *M. marinum.* Consequently, the extent to which this property pervades within clinical isolates, and the relevance to mycobacterial pathogenesis, remains incompletely understood. We therefore examined the intracellular niche preferences, in infected macrophages, of eight Mtb isolates by transmission electron microscopy (TEM). Surprisingly we found that individual strains displayed a variable capacity to escape from phagosomes, with strain-specific differences extending to both quantitative and kinetic aspects of the process. Especially notable in this context was that phagosome escape was initiated very early in some cases, occurring either soon after–or even concurrently with–phagocytic uptake of the bacteria. Inhibition of this process compromised intracellular bacterial survival, implying that early escape was necessitated for these strains on account of a diminished capacity to tolerate phagosomal stresses. Translocation then served as a ‘virulence-rescue’ mechanism since cytoplasmic localization favored a more effective suppression of autophagy in the host macrophage. Thus, our studies uncover an additional dimension to the adaptation strategies exploited by Mtb, for survival in the host macrophage.

## Results

### Mtb strain-dependent variations in intracellular niche preference

**Figure 1:**
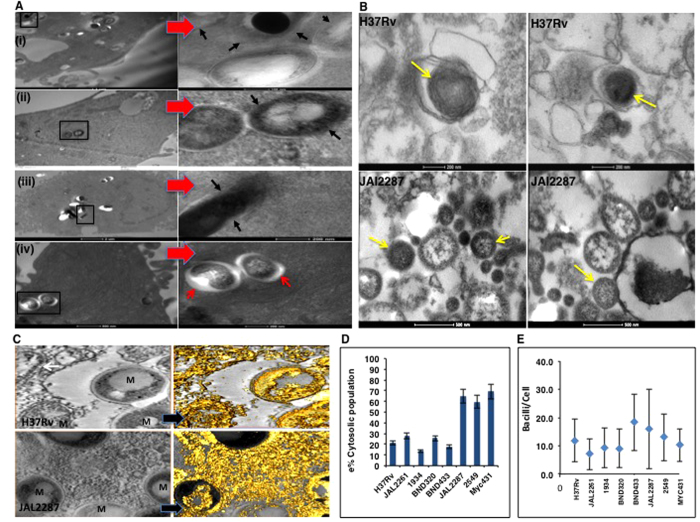
Mtb strain-dependent differences in intracellular localization. (**A**) Representative Transmission electron micrographs of infected cells showing Mtb existing in absence of a vesicle (i), (ii), (iii) in comparison to Mtb in vesicle structures (iv). Black arrows emphasize contiguity of the bacterial wall with the macrophage cytoplasm. Distinct vesicles (marked by red arrows), (iv) for certain vesicle associated strains of Mtb. Left side panels are low power images of the macrophage showing Mtb infection. Right side panels are blown up images of adjacent images (black box) showing Mtb in vesicle free and vesicle associated forms. Magnification: 2 um and 200 nm (i), 500 nm and 20 nm (ii), 2 um and 200 nm and (iii) and 200 nm and 500 nm (iv). More than 100 cells were investigated from multiple experiments. (**B**) Representative transmission electron micrographs showing Mtb particles (yellow arrows) in phagosomal preparations. The two upper figures show bacteria that are closely associated with vesicular membranes. The lower micrographs show free bacteria as identified by yellow arrows. More than 100 cells were viewed from 3 separate experiments (**C**) 3D Visualization of intracellular bacteria using electron tomography. Top panel shows a vesicle-enclosed H37Rv bacterium (top, left panel), which was reconstructed using isocontour volume rendering (top, right panel). The bottom panel shows a cytoplasmic JAL2287 bacterium (left panel), where a similar volume rendering shows clear intracellular electron dense bodies and continuity with the cytoplasmic mass (right panel). “M” refers to Mtb. (**D**) Relative proportion of intracellular bacteria that were cytosolic in cells infected for 24 hr with each of the strains. Values were determined by counting bacteria in >100 cell sections (mean ± S.D.). Bacillary load was enumerated after staining of the cells with Ziehl-Neelsen stain, and results are given in panel E as the mean (±S.E.) value for >200 cells.

We studied here a group of eight virulent Mtb strains, which in addition to H37Rv also included the clinical isolates JAL2287, BND433, BND320 (all of CAS lineage), JAL2261, 1934 (both of the Manu clade), MYC431 (Beijing strain), and 2549 (miscellaneous). These isolates have been described earlier^12^,^13^. PMA-differentiated THP-1 cells were infected with each of these strains and intracellular localization of the bacteria was examined 24 hrs later by TEM. Five of the strains showed evidence of the bacilli being primarily localized within membrane-bound vesicles with only a small fraction being detected free in the cytoplasm. Representative TEM for the same is shown in Fig. 1A-(iv) and Fig. S1A. In contrast, the predominant population of JAL2287, 2549, and MYC431 bacteria were not present in membrane-enclosed compartments but, rather, was localized to the cytosol (Fig. 1A-(i),(ii),(iii), Fig. S1B). Here, bacteria lacked the characteristic electron translucent zones or were unassociated with any such vesicular structure, as suggested by the contiguity of the bacterial cell wall with the cytoplasm (Fig. 1-(i),(ii),(iii), Fig. S1B).

This marked distinction that segregated the strains into two groups was somewhat surprising. To verify this further we prepared lysates from H37Rv- and JAL2287-infected cells by using a protocol that leaves the membrane components intact^8^. TEM examination of these lysates revealed that, while the H37Rv bacteria were contained within vesicles, those of JAL2287 indeed remained free of any such bounded compartments (Fig. 1B). Unambiguous confirmation was then sought through electron tomographic 3D imaging of representative fields in sections from H37Rv- and JAL-2287-infected, cells. Electron tomography provides a powerful volume rendering of ultrastructural components and has been extensively used for 3D visualization of fine structure, as well as host-pathogen interaction events^14^. Analysis of the tomogram data through isovolume rendering in the two instances clearly confirmed the cytoplasmic existence of JAL2287 without any artifacts (Fig. 1C, lower panel). Moreover, the reconstruction revealed no connectivity of the JAL2287 bacterium through any specialized structure of the host cytoplasm but, rather, highlighted its presence in the free form (Fig. 1C, lower right panel). In contrast, analysis of the tomogram data for H37Rv only served to further reinforce its vesicle-enclosed nature (Fig. 1C, top panel).

Figure 1D shows the distribution between membrane-free versus vacuolar bacteria that was obtained for the different strains in THP-1 cells, whereas Fig. 1E compares the bacillary load per cell in each case. No correlation was detected between these two features, which is consistent with earlier findings that phagosomal escape of bacteria does not at least entirely depend on the infection load^11^,^15^.

**Figure 2:**
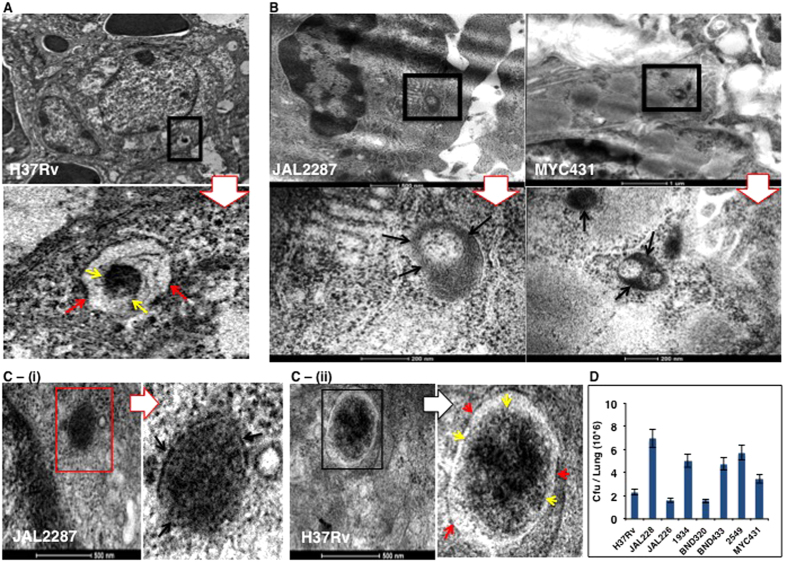
Strain-distinctive intracellular niche preferences are also retained *in vivo.* Groups of eight mice each were infected through the aerosol route with the individual Mtb strains (100–150 bacilli/lung^13^). At 15 days later these mice were sacrificed and the lungs harvested. Lungs from two mice in each group were taken for TEM imaging and panels (**A**,**B**) shows the representative micrographs obtained. Panel (**A**) shows two representative images of lung cells containing phagosome-enclosed bacteria for the H37Rv (marked by black box) where phagosomal membranes (marked by red arrows) surrounding the bacteria can be distinguished from the bacterial cell wall (marked by yellow arrows). Lower image shows the blown up Mtb structure, boxed (black) in the upper low power shot showing the lung field. Magnification–1 um. 70–100 cells were observed in lung sections for all lung investigations. Panel B confirms cytosolic residence of JAL2287 and MYC431 bacteria in the lung cell field (marked by black box). Upper panels represent low power images showing the lung macrophages followed by their respective high power shots in the lower panels. Continuity of the bacterial wall (black arrows) of the Mtb (Black box) from the low power is shown in the images. Magnification–500 nm and 1 um (upper) and lower–200 nm in both cases. Panel (**C**-(**i**)) and (**C**-(**ii**)) show the blown up images showing distinct absence and presence of vesicle structure in lung macrophages, for the indicated strains respectively. Yellow arrows mark the bacterial wall, whereas, the vesicle membrane is marked by red arrows. Field from left side images of both panels (marked by boxes-red and black) are blown up to show the Mtb structures in the adjacent panels Magnification: 500 nm. Lysates were generated from lungs of the remaining six mice per group, and plated for determination of the Cfu values (**D**). Values are the mean ± S.D.

To confirm that the observed differences in intracellular niche preferences truly represented intrinsic properties of the individual strains, rather than a derivative of the host cell line used, we next infected mice with each of the Mtb strains. At 15 days later these mice were sacrificed and the lungs removed for subsequent processing and examination by TEM. We specifically monitored the infected alveolar macrophages, based on morphological criteria, and the results thus obtained reaffirmed our findings in THP-1 cells. That is while the H37Rv, JAL2261, BND433, BND320 and 1934 bacilli remained enclosed within vacuoles (Fig. 2A, Fig. S1C), between 55–67% of the JAL2287, MYC431 and 2549 bacteria were found to be extra-vacuolar (Fig. 2B, Fig. S1D). This is highlighted by the representative high-magnification image for JAL2287 where the continuity of bacterial cell wall with the cytoplasm of lung macrophage is clearly evident, as compared to the situation for the vesicle-enclosed H37Rv (Fig. 2C-(i), C-(ii)). Importantly, we could again confirm that this distinction in sub-cellular localization was not due to differences in bacterial loads. A parallel enumeration of bacterial counts in lungs of the corresponding mice revealed an overlap in the range of CFU values obtained for the two groups (Fig. 2D).

Thus our above results highlight the marked differences in apparent proficiencies exhibited by the individual Mtb isolates, in terms of translocating from phagosomes to the cytosol. Since only single time point observations were made however, it was unclear whether these differences reflected quantitative differences in the capacity to translocate, or, variations in the timing at which the individual strains initiated this process. In this context our finding that JAL2287, 2549, and MYC431 bacteria were already localized primarily in the cytoplasm by 24 hr after THP-1 cell infection (Fig. 1B is especially significant. It contrasts with earlier suggestions that mycobacteria are generally contained within endosomes in the initial stages of infection^10^,^11^,^15^.

### Rapid initiation of cytoplasmic translocation in JAL2287-infected cells

**Figure 3:**
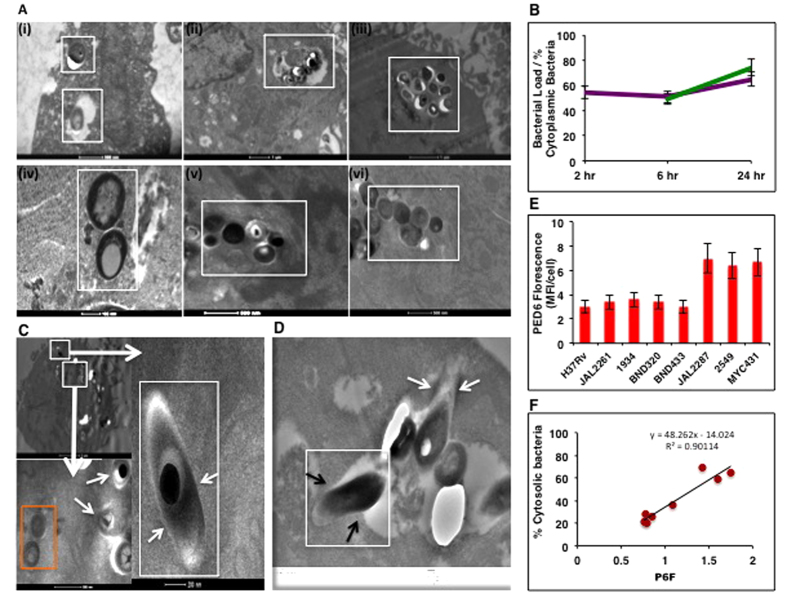
Time-course study of THP-1 cells infected with H37Rv and JAL2287. (**A**) Representative images of cells infected with either H37Rv (i–iii) or JAL2287 (iv–vi) and a TEM analysis was performed at various times thereafter. Results shown are for 2 (i, iv), 24 (ii, v), and 36 (iii, vi) hours p–i. Magnification, 500 nm for (i), (v) and (vi); 1 nm for (ii) and (iii), 100 nm for (iv). >200 cells were observed from multiple infections. (**B**) Comparison of time-dependent changes in bacterial load (green line) versus the corresponding changes in the proportion of cytoplasmic bacteria (purple line) in JAL2287-infected cells. The Y-axis gives either the bacterial load (cfu/well (x10^3^)), or the cytosolic bacteria as a percent of the total number of bacteria/cell. (**C**) A composite of images reflecting both cytoplasmic (white box) and vesicle-associated bacteria in cells after a 1 hr infection period with JAL2287 (top left panel, magnification–2 um). The boxed regions are also shown at a higher magnification (bottom and right panels, magnification 500 and 200 nm respectively) to confirm their cytoplasmic localization. The “bulls eye” appearance characteristic of Mtb is also evident here. >200 cells were observed from multiple experiments. (**D**) Another phagosome from this experiment (magnification, 100 nm) where one of the bacteria appears to be in the “entry mode” (white arrows), while another appears exiting the phagosome (white box), with its membrane fused to the phagosome (black arrows) membrane. Non-specific debris that may be of bacterial origin can be seen. (**E**) PED-6 fluorescence was monitored at 24 hr p–i. by confocal microscopy in infected THP1 cells. The mean fluorescence intensity (MFI)/cell of PED6 hydrolysis/cell is expressed in infected cells, values are expressed as a ratio over the corresponding value obtained for uninfected cells (>200 cell observed, ± S.D., n = 3). Panel **F** shows the results of a linear regression analysis performed between the values of PED-6 fluorescence intensities in panel **E** (PF6), and the percent cytosolic bacteria obtained in Fig. 1D. The positive correlation implies a link between host cytosolic phospholipase A2 activation and phagosomal escape of Mtb.

To further probe the possible occurrence of phagosome escape in the early stage of infection, we next studied cells infected with JAL2287 for periods ranging from 2 to 48 hours. For the purposes of comparison, a parallel set of H37Rv-infected cells was also included in these experiments. Surprisingly, extra-vacuolar JAL2287 bacteria were evident even as early as 2 hrs after infection (Fig. 3A). Indeed, inspection of >200 bacteria at each of the time points revealed that near peak levels of cytosolic bacteria was already achieved by this time point, with only a minor change thereafter (Fig. 3B). This was in spite of the fact that the cellular bacterial load increased significantly over this period (Fig. 3B). In contrast to this, H37Rv-infected cells predominantly displayed vacuolar bacteria across all the time points studied (Fig. 3A).

To resolve the above observation further we next generated sections from cells exposed to JAL2287 for only 1 hr, and analyzed them by TEM. Representative images are shown in Fig. 3C,D. It is obvious from Fig. 3C that, although vesicle-localized bacteria were present, several cytosolic bacteria also already appeared by this time. Figure 3D further elaborates this observation by depicting a phagosome where one of the bacteria is partly extruded out of the vesicle, with its membrane now fused to the phagosome membrane. Thus the results in Fig. 3C,D together suggest that, at least for JAL2287, phagosomal escape likely occurs in tandem with the infection process.

### Phagosomal escape is mediated through cytosolic phospholipase A2 activation

Previous studies have established that cytosolic translocation of mycobacteria is mediated by ESAT-6^9^,^10^,^11^,^15^. The membrane lysing activity of the protein has been implicated in this function, and more recent studies have delineated the functional domain to reside at its C-terminal end^9^. Although ESAT-6 is undoubtedly essential it alone, however, was not sufficient to explain the observed strain-specific differences in vacuolar versus cytosolic distribution of the bacteria. This was because, as previously shown^13^, all of these strains secrete ESAT-6 in the host cell milieu. Therefore, an additional pathway likely complemented with ESAT-6, to regulate strain-specific variability in the extent and kinetics of phagosomal escape. Such an inference would also be consistent with earlier observations that even BCG, which does not express ESAT-6, shows a limited capacity for permeabilizing phagosomes^16^.

The cytoplasmic phospholipase A_2_ (cPLA2) enzymes play a critical role in both phagosomal trafficking, and export of cargo from the various endocytic compartments^17^,^18^,^19^. These enzymes cleave the sn2 acyl bond of phospholipids, releasing arachidonic acid and lysophospholipids. Previous studies have shown that cPLA_2_ expression was enhanced in Mtb-infected macrophages^20^, and that recruitment of the subsequently derived arachidonic acid into the 5-lipoxygenase-dependent pathway inhibited macrophage apoptosis and cross-presentation of Mtb antigens by dendritic cells^21^. In the context of the present study though, cPLA_2_ has also been shown to permeabilize the endosomal membrane in Mtb-infected macrophages^22^. Therefore, to test whether such activities may additionally contribute to Mtb cytosolic translocation we employed PED-6, a dye-labeled substrate for PLA_2_. PED-6 is a glycerophosphoethanolamine probe with a BIODIPY dye-labeled acyl chain at the sn2 position, and with the head group modified with a dinitrophenyl quencher. Cells infected with each of the eight strains were loaded with a liposomal preparation of the PED-6 probe as described earlier^22^ and cPLA_2_ activation was determined by intensity of the resulting fluorescence signal. The latter was a result of the unquenching of BIODIPY fluorescence, due to phospholipase (cPLA2)–dependent cleavage of the sn2 acyl bond.

**Figure 4:**
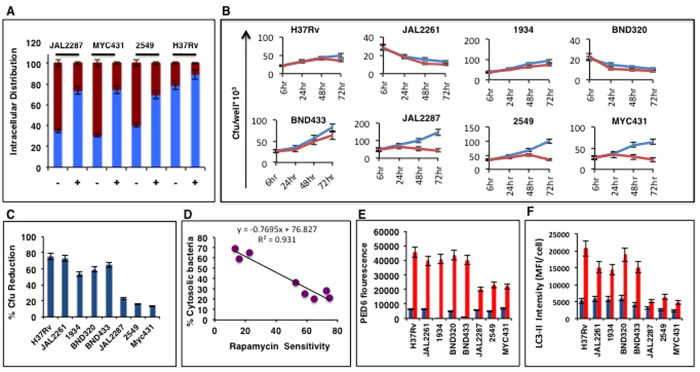
Cytosolic translocation of Mtb is facilitated through PLA_2_ activation. (**A**) THP-1 cells were infected with the Mtb strains either in the absence (−) or presence (+) of the PLA_2_ inhibitor AACOCF3. At 24 hrs later, the cells were fixed and processed for TEM. The percent of intracellular bacteria distributed between the endosomal (blue) and cytosolic (red) compartments was determined by averaging the values from more than 100 cell sections in each case. In panel (**B**), Mtb-infected THP-1 cells were either left untreated (blue line) or treated (red line) with AACOCF3. At indicated times, cells were lysed and the intracellular bacillary load determined in terms of the cfu counts. (**C**) Infected THP-1 cells were either left untreated or treated with rapamycin for 6 hrs. Lysates were then generated at 60 hrs p–i, and the bacterial load determined in terms of the cfu counts. Results are expressed as a percent reduction in cfu values in rapamycin treated cells, compared to those in the untreated cells in each case (mean ± S.D., n = 3). (**D**) Results of a linear regression analysis performed between the data in panel (**C**) (rapamycin sensitivity), and that in Fig. 1D (% Cytosolic bacteria). In panel (**E**), cells infected with the indicated Mtb strains for 24 hr were either left untreated (blue bars), or treated with rapamycin for 6 hrs (red bars). Subsequently, LC3-II levels were measured by confocal microscopy and bars show mean (±S.E.) fluorescence intensity per cell (MFI/cell). Values were averaged over 80–100 cells in each case. In Panel (**F**), Mtb infected cells were either left untreated (blue bars) or treated with Interferon gamma for 48 hrs (red bars). LC3-II levels were measured by confocal microscopy and bars show mean fluorescence intensity per cell (±S.E) after averaging the values obtained for over 100 cells in each case.

Although some degree of cPLA_2_ activation was evident in all cases, significantly higher levels were obtained in cells infected with the three strains that also displayed a bias towards cytosolic residence (Fig. 3E). Notably, a regression analysis between PED-6 fluorescence intensity and the proportion of cytoplasmic bacteria obtained in the differently infected cells yielded an excellent positive correlation (Fig. 3F), implying a link between phospholipase (cPLA2) activation and phagosomal escape of Mtb. This interpretation was further supported by the results of our subsequent experiments employing arachidonyl trifluoromethyl ketone (AACOCF3), an inhibitor of cPLA_2_^23^,^24^. Mtb infection of cells in the presence of AACOCF3 led to the expected suppression of PED-6 fluorescence by 24 hr (Fig. S2A). Significantly, this treatment also resulted in a marked reduction in cytosolic population of the JAL2287, 2549, and MYC431 bacteria (Fig. 4A). This reduction was not due either to a loss in host cell viability, or any significant reduction in the cellular bacterial load (see Fig. 4B). Instead, it appeared to derive from an enhanced restriction of bacilli within the phagosomal compartment (Fig. 4A). Thus, at one level, these findings support that cPLA_2_ activity also contributes towards regulating phagosomal escape of Mtb. In addition, Fig. 3F also supports that the observed strain-specific differences in extent of translocation likely derive from corresponding differences in ability of the Mtb strain to activate host cPLA2 in the intracellular milieu.

As opposed to the effects of short-term treatment, extended exposure to AACOCF3 of cells infected with either of the cytosol-preferring strains (JAL2287, 2549, MYC431) led to a progressive reduction in bacterial counts in all cases (Fig. 4B). This effect, however, was selective since the bacillary load in cells harboring any of the other, vesicle-localized, isolates remained relatively insensitive to the treatment (Fig. 4B). We subsequently also confirmed that AACOCF3 did not influence viability of either bacteria when grown in extracellular cultures, or of the infected host cells (Fig. S2B,C). Rather, a TEM analysis revealed that AACOCF3-treatment of infected cells caused an increased vesicular degradation of the cytosol-preferring, but not the endosome-localized, Mtb strains (Fig. S2D,E). Thus, taken together with the findings in Fig. 4A, these results indicate that a sustained prevention of phagosomal escape renders the cytosol-preferring strains more vulnerable to the anti-bacterial mechanisms of the host macrophage.

### Cytosolic residence confers resistance to autophagy

Intriguingly, treatment of infected cells with rapamycin produced distinct effects on the vesicular- versus the cytosol-resident groups of Mtb strains. Whereas intracellular viability of the former was markedly compromised, the latter group proved to be significantly more resistant (Fig. 4C). Rapamycin is a bacterial macrolide that inhibits the mammalian target of rapamycin (mTOR) and, thereby, stimulates autophagy^25^. Studies from several laboratories have established that autophagy represents a key anti-mycobacterial mechanism of the macrophage, and the ability to suppress this process has been intricately linked to mycobacterial virulence^12^,^26^,^27^. Thus our finding in Fig. 4C suggests that the cytosol-preferring Mtb strains gain in virulence function by acquiring resistance to autophagy. The interpretation that cytosolic residence confers resistance to autophagy is additionally supported by the strong negative correlation seen between the proportion of cytosolic bacteria obtained for the different strains, and their relative insensitivity to rapamycin treatment (Fig. 4D). In subsequent experiments we found that rapamycin-dependent activation of autophagy was attenuated to a greater extent in cells infected with either of the cytosol residing Mtb strains, as opposed to those displaying a bias towards endosomal localization (Fig. 4E, Fig. S3). To further verify that intracellular localization of Mtb differentially influences autophagic responses of the host cell, we also tested under the physiologically more relevant condition of IFN-γ stimulation. Several studies have documented a central role for IFN-γ in the host immune response to Mtb infection^28^,^29^,^30^. Among other effects, the reported mode of action of this cytokine includes activation of autophagy and phagosome maturation in the infected macrophage^31^,^32^. Thus, infected cells were stimulated with IFN-γ and then monitored for autophagy induction. The results obtained are shown in Fig. 4F. It is evident here that, while cells infected with either of the vesicle-localized Mtb strains displayed a strong autophagy response the effect of IFN-γ was–however–significantly attenuated in cells harboring the cytosol-preferring bacteria. Importantly though in the latter instance, sensitivity to IFN-γ was markedly improved when cytosolic translocation of the bacteria was prevented by treatment of cells with AACOCF3 (Fig. S4). Thus, while the mechanism remains unknown, our cumulative findings confirm that cytosolic localization of Mtb is associated with an enhanced capacity for suppressing activation of host cellular autophagy.

## Discussion

Our study provides an added perspective to the accumulating support for Mtb translocation from phagosomes to the cytosol of infected macrophages. Contrary to the prevailing notion that translocation always occurred several days post-infection, we found that the time course of this process was in fact strain dependent. Thus, in some strains, cytosolic translocation was evident within hours after macrophage entry. Indeed in the case of JAL2287, phagosomal escape appeared to be co-initiated with the infection process and a dominance of the cytoplasmic over the endosomal pool of bacteria was evident by as early as two hours of infection. Also notable is our finding that ESAT-6 alone could not account for the observed differences in translocation competency of the various Mtb strains. Instead, this latter feature showed a strong correlation with the degree of cPLA_2_ activity that was induced within infected cells. Thus, host cPLA_2_ activity likely complements ESAT-6 function to regulate phagosomal escape of Mtb. The basis for differential cPLA_2_ activation by the individual Mtb strains, however, awaits future clarification.

Intriguingly, for the Mtb strains that preferentially localized to the cytoplasm, prevention of phagosomal escape through lipase inhibition rendered them vulnerable to killing by the host macrophage. In contrast, cPLA_2_ inhibition had only a marginal effect on intracellular viability of those Mtb strains that were primarily restricted to the endosomal compartment. At least based on our preliminary findings, this distinction in sensitivity to lipase inhibition appeared to reflect a differential capacity between these two groups of strains to withstand phagosomal stresses. Consequently, it is tempting to speculate that early translocation into the cytoplasm presents an alternate survival mechanism for such strains that are less adapted to the endosomal environment. While this proposition is yet only a hypothesis and requires verification, the possibility that cytosolic translocation provides relief from microbicidal mechanisms of the macrophage has been previously suggested^8^,^15^.

Earlier reports have indicated that, relative to the phagosome, the cytoplasm provides a more conducive environment for mycobacterial replication^11^,^33^. Further, by^8^ subsequently inducing necrotic death of the host cell, cytosolic translocation has also been suggested to facilitate escape of the bacteria from the intracellular environment altogether^10^,^34^. In addition to these properties though, our study identified yet another pro-survival trait that was acquired by the bacilli as a result of cytosolic translocation. In comparison with Mtb strains that predominated in the endosomes, those biased towards cytosolic residence were found to be significantly more adept at inhibiting the activation of autophagy in the host cell. Given that autophagy serves as a key pathway for clearance of intracellular bacteria by macrophages^12^,^26^,^27^, cytosolic translocation could well then constitute a ‘restoration-of-virulence’ strategy for those Mtb strains that are less adapted to survive within phagosomes. That is, the susceptibility resulting from an inherent deficit in the latter property was recompensed by a concomitant gain in resistance to autophagy.

The broad range of bactericidal responses unleashed by the macrophage requires the pathogen to acquire a portfolio of countermeasures, in order to survive and persist in the intracellular milieu. Such countermeasures include prevention of phagosomal maturation, suppression of oxidative responses, prevention of apoptosis, accession of nutrients, and capacity to enter into a non-replicative state among others^35^. In such a scenario then, ‘pathogen fitness’ could likely represent a combinatorial outcome of the relative contribution from these individual defense pathways. Our present study is especially relevant in this context as it uncovers an additional level of plasticity to the adaptation mechanisms resourced by Mtb, for surviving within macrophages.

## Methods

### Infection, Treatment, and CFU Determination

Infection of THP-1 and HuMϕ cells with at a multiplicity of infection of 10:1, and determination of CFUs were done as described earlier^12^,^13^. Where required, AACOCF3 was added at a final concentration of 25 μM and Rapamycin at 5 μM. THP-1 cells were originally obtained from “ATCC” and maintained in our laboratory. For mice experiments, animal were obtained from the small animal facility of ICGEB and were infected with Mtb strains through aerosol exposure by delivering between 100–150 bacteria per lung. We confirmed the comparable bacterial uptake in all the groups by measuring the cfu in lungs of a parallel set of animals (3/group) at 24 hrs after infection. Two weeks from infection the mice were sacrificed and lungs were removed. Lungs from two mice in each group were taken for TEM imaging and remaining were plating for cfu determination as previously reported^12^,^13^.

### Transmission electron microscopy

Infected cells, or pieces of infected lungs were washed with sodium cacodylate buffer (pH, 7.4) and fixed in 3% glutaraldehyde. Cells were then osmicated and dehydrated prior to embedding in EPON 812 resin^36^. Polymerized blocks were cut into 80–100 mm ultrathin sections and collected on 200 or 400 mesh size copper grids. Sections were stained with Uranyl acetate (10 min–1 hr), followed by Reynolds lead citrate (2 min) and then imaged under 80 and 100 KeV operating voltages of a Tecnai 12 Biotwin transmission electron microscope (FEI Co, The Netherlands). Images were captured using a side mounted 2 k × 2k CCD camera (Megaview III, SIS Germany). Size calibration and morphometry was done using the TIA image analysis software (FEI Co, The Netherlands).

Extensive control imaging was done to ensure that the images collected were free of artifacts. This included monitoring for the natural preservation of nucleus, nuclear envelope, mitochondria and other cyto-organelles. Further, an adequate number of cell profiles and imaging fields were seen before any interpretation was made. Finally, we also ensured that our interpretations were based on published literature and standard reference material on ultrastructural pathology. For bacterial detection and enumeration, only those displaying morphologically relevant features as described in the literature were considered.

### Electron tomography

The stained grids imaged under 200 KeV operating voltage in a Tecnai-120 transmission electron microscope. Single axis serial tilt tomograms were recorded from +50° to −55° using a 2° tilt interval and 0.5° interval in high range were collected. The acquisition was done using a automated tomography acquisition software (XPLOR 3D ^TM^ suite, FEI, Co, The Netherlands). The goniometer stage centering, eucentric height adjustment, holder calibrations, autofocus function adjustments, cross-correlation feature tracking, were all automated and controlled by the automated-tomography software. Tomogram series were stored as MRC files in a networked Terastation (Buffalo computers USA). Tomogram sets were recorded at magnification of 200 nm.

For image processing and volume reconstruction, the acquired tomogram sets were subsequently processed using the Inspect 3D software (FEI Co., The Netherlands), stacks aligned and de-noised. The volume reconstructions of areas of interest were carried out using both Weighted Back Projection (WBP) and SIRT method with 20 iterations. Visualization was done using the AMIRA 5.0 software (AMIRA, France).

### Confocal microscopy

Cells stained with either PED-6 or with anti-LC3-II were imaged on a Nikon Eclipse Ti-E laser scanning confocal microscope equipped with 603/1.4 NA PlanApochromat DIC objective lens. Nuclei were counterstained with DAPI. Images were acquired and analyzed with Image-Pro Plus version 6.0.

### Ethics Statement

Animal experiments were conducted in the DBT-funded TACF facility at ICGEB and in compliance with guidelines created and approved by ICGEB animal ethics committee. The Protocol for animal use and care was reviewed and approved by the Institutional Animal Ethics Committee (IAEC) of International Centre of Genetic Engineering and Biotechnology, New Delhi (ICGEB, New Delhi). Experiments adhered to the guidelines of Committee for the Purpose of Control and Supervision of Experiments on Animals (CPSEA), Government of India, for Laboratory Animal Facilities. The reference number for the approval (IAEC number) is ICGEB/AH/2013/1/IMM32.

## Additional Information

**How to cite this article**: Jamwal, S. V. *et al.* Mycobacterial escape from macrophage phagosomes to the cytoplasm represents an alternate adaptation mechanism. *Sci. Rep.* **6**, 23089; doi: 10.1038/srep23089 (2016).

## Supplementary Material

Supplementary Information
