# Supplementary material for: Mycobacterial escape from macrophage phagosomes to the cytoplasm represents an alternate adaptation mechanism
Source: Sci Rep. 2016 Mar 16;6:23089. doi: 10.1038/srep23089 (PMC4793295; doi:10.1038/srep23089)
Supplement: Supplementary Information [file srep23089-s1.pdf]

Mycobacterial escape from macrophage phagosomes to the cytoplasm represents an alternate adaptation mechanism

Shilpa V. Jamwal[1,4], Parul Mehrotra[1,4], Archana Singh[2], Zaved Siddiqui[1] , Atanu Basu[3,5], and Kanury V.S. Rao[1,5]

[1]Immunology Group
International Centre for Genetic Engineering and Biotechnology
Aruna Asaf Ali Marg, New Delhi – 110067, India

[2]Systems Biology Group
Institute of Genomics and Integrative Biology
Mall Road, New Delhi – 110007, India

[3]National Institute of Virology
Dr. Babasaheb Ambedkar Road
Pune – 411001, India

[4]Equal contribution
[5]Correspondence: basua@icmr.org.in; kanury@icgeb.res.in

Supporting Information

Figure S1: Confirmation of Mtb strain-dependent differences in intracellular niche preference.

A: Representative transmission electron micrographs of infected cells showing Mtb bacteria in association with cytoplasmic vesicles (white arrows). Two images for each indicated strain are shown. Bacterial morphology was largely consistent and did not show degradation. Bacterial cell wall (yellow arrow head) can be seen distinct from vacuolar membrane. Magnification, 500 nm for H37Rv (i) and BND320 (i); 200 nm for H37Rv (ii), 1934 (i) and (ii), JAL2261 (i) and (ii); 100 nm for BND320 (i), BND433 (i) and (ii).

B: Four representative images for cytoplasmic existence of bacteria in cells infected with each of the indicated strains. White arrows emphasize contiguity of the bacterial membrane with the macrophage cytoplasm. Magnification 200 nm for JAL2287 (iii), 2549 (iii) and (iv); 100 nm for all the remaining panels. Some images have been cropped to highlight the region while maintaining the original scale.

C-D : Groups of eight mice each were infected through the aerosol route with the individual Mtb strains (100-150 bacilli/lung [13]). At 15 days later these mice were sacrificed and the lungs harvested. Lungs from two mice in each group were taken for TEM imaging and panels C and D shows the representative micrographs obtained. Panel C shows two representative images of lung cells containing phagosome-enclosed bacteria for the indicated strains (marked by black boxes) where phagosomal membranes (marked by yellow arrows) surrounding the bacteria can be distinguished from the bacterial cell wall. Magnification: 500nm. Panel D confirms cytosolic residence of Mtb in the lung cells (marked by black boxes). Magnification is 200 nm in both cases.

Figure S2. Intracellular viability of the cytosol-preferring Mtb strains is sensitive to $cPLA_2$ inhibition.

A: Mtb-infected, and PED-6-labeled THP-1 cells were cultured either in the absence (blue bars) or presence (red bars) of AACOCF3 (25uM). At 24 hrs later, >200 cells were imaged by confocal microscopy and the Mean florescence intensity (MFI) per cell was determined (± S.D., n = 3).

B: JAL2287 was cultured in 7H9 medium Effect of on free cultures of Mtb was determined by measuring the Optical density of the culture media over five days in either the absence (blue line) or the presence (red line) of AACOCF3 (25uM). Optical density (600 nm) of the cultures was measured daily over a subsequent 5-day period and the results are presented here.

C: AACOCF3 does not affect viability of infected THP-1 cells. Uninfected cells (UI), or cells infected with the indicated Mtb strains were cultured either in the absence (blue bars) or presence (red bars) of AACOCF3 and the extent of cell death 24 hrs later was determined by using the GFP-certified Apoptosis/Necrosis detection kit (Enzo Life Sciences).

D: THP-1 cells were infected with Mtb strains either in the absence (blue bars) or presence (red bars) of AACOCF3, and were then processed for TEM at 48 hrs post infection. The sections were analyzed for the presence of Phagosome – Lysosome (P-L) structures suggestive of bacterial degradation. The number of P-L structures was enumerated in more than 100 cell sections, and the average values (± S.E.) per section are shown here. Data are a representative of two separate experiments.

E: TEM images of representative fields of sections from JAL2287-infected cells treated with AACOCF3. Phagosome – lysosome structures (boxed) containing electron dense deformed bacterial debris, which is suggestive of increased bacterial degradation, can be observed here.

Figure S3. Induction of autophagy in infected cells upon infection with vesicular versus cytosolic strain.

Panel shows representative confocal microscopic images comparing the level of LC3-II (red) in H37Rv and JAL2287-infected cells either with (+) or without (-) rapamycin treatment.

Figure S4. The intracellular localization of Mtb dictates the host cellular autophagy response.

A: Cells were infected with the indicated Mtb strains and maintained in either absence (blue bars) or presence of AACOCF3 (red bars). All cells were stimulated with Interferon gamma post infection. LC-3II levels were measured at 48hrs p-i by confocal microscopy and bars show mean fluorescence intensity per cell (± S.E) after averaging the values obtained for over 100 cells in each case.

B: Mtb (strains) infected THP-1 cells were transfected with either control (GFP specific) or cPLA2 specific siRNA. 72 hours later cells were lysed and lysates were plated for cfu determination as previously described [12]. Bars represent cfu counts for the control (blue) compared to target siRNA treated cells (red) (Mean± S.D., n=3).

Figure S1

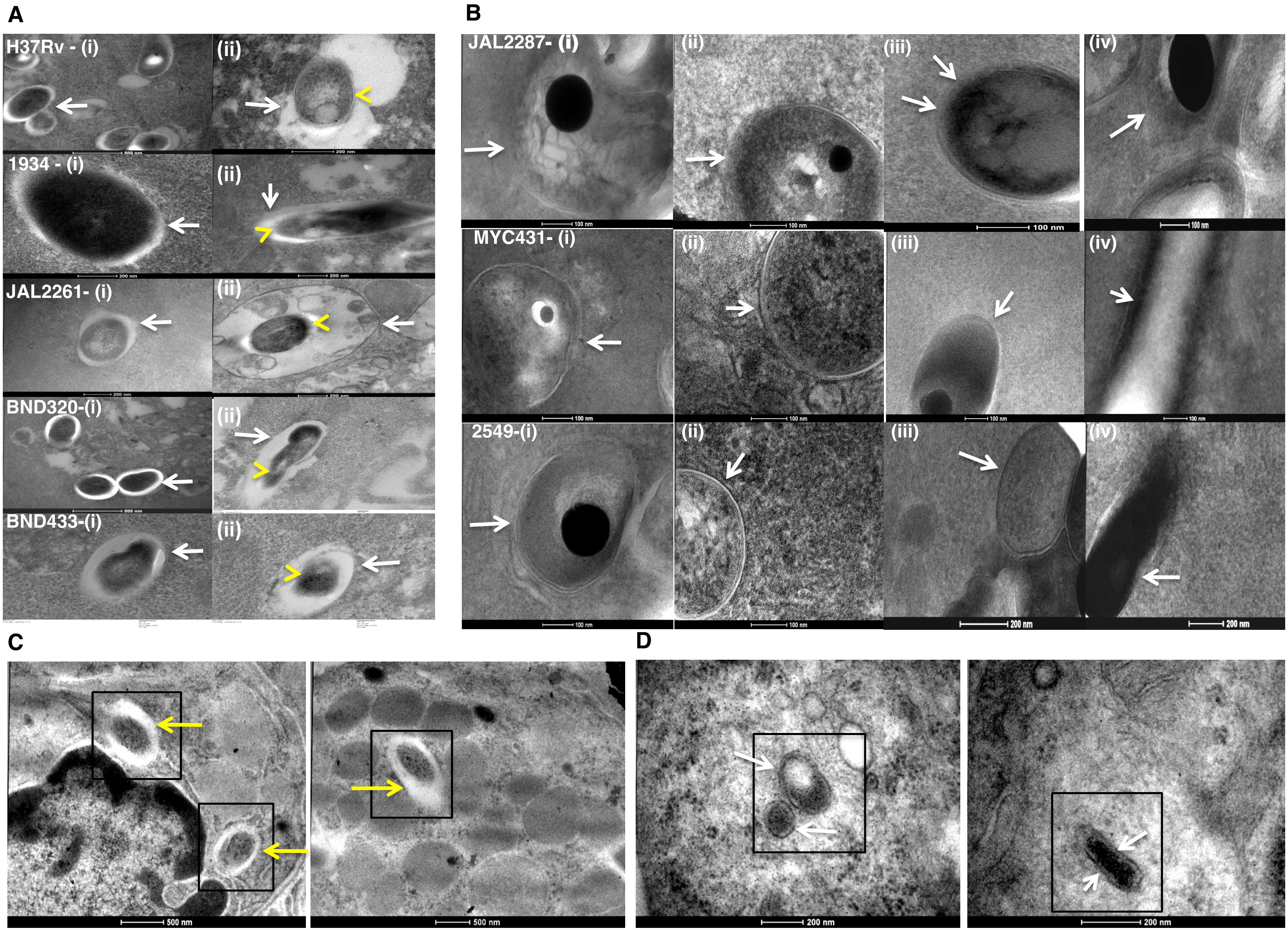
A
H37Rv - (i)
(ii)
1934 - (i)
(ii)
JAL2261- (i)
(ii)
BND320-(i)
(ii)
BND433-(i)
(ii)
B
JAL2287- (i)
(ii)
(iii)
(iv)
MYC431- (i)
(ii)
(iii)
(iv)
2549-(i)
(ii)
(iii)
(iv)
100 nm
200 nm
C
500 nm
D
200 nm

Figure S2.

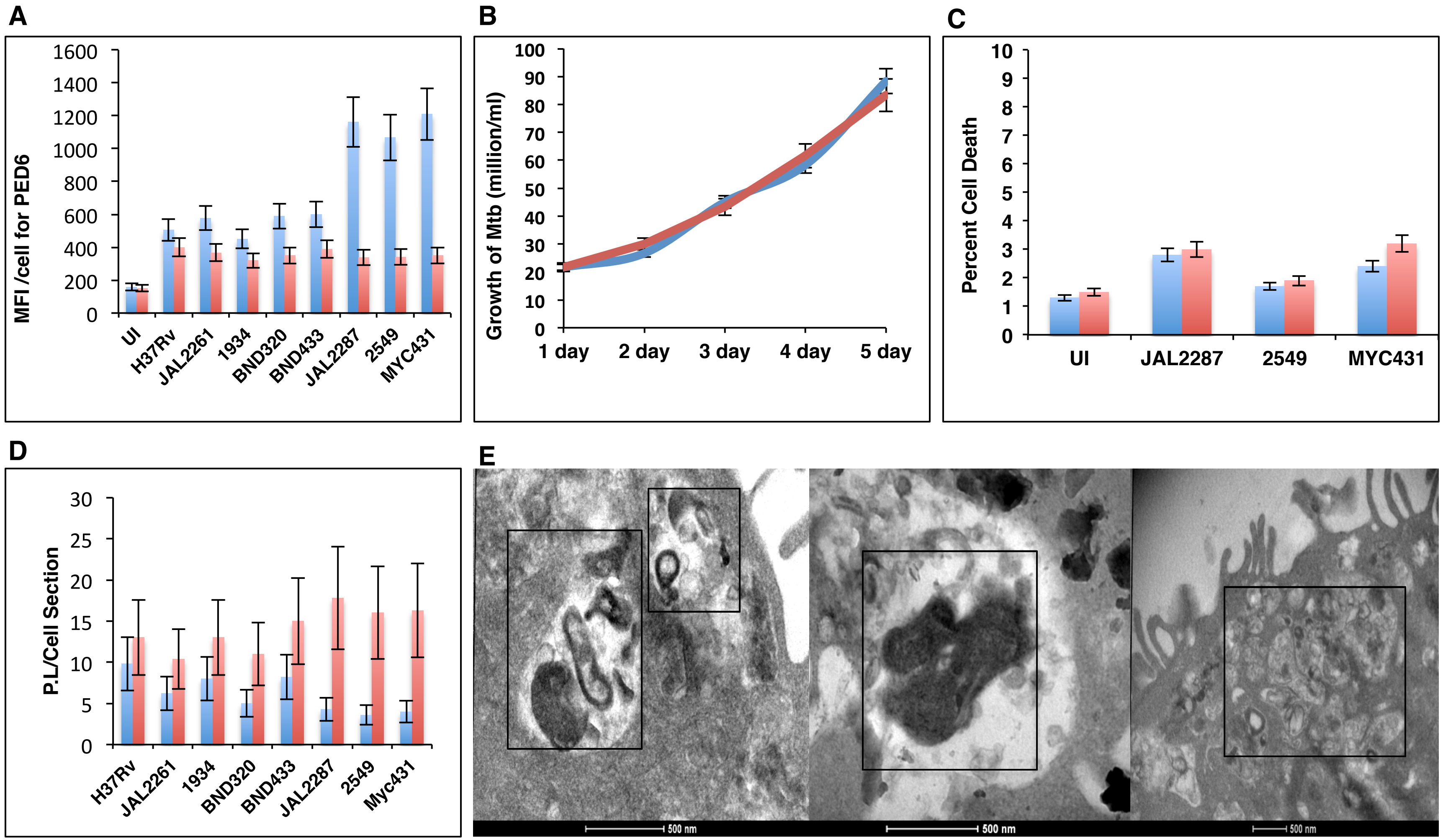
A
MFI /cell for PED6
1600
1400
1200
1000
800
600
400
200
0
UI
H37Rv
JAL2261
1934
BND320
BND433
JAL2287
2549
MYC431
B
Growth of Mtb (million/ml)
100
90
80
70
60
50
40
30
20
10
0
1 day
2 day
3 day
4 day
5 day
C
Percent Cell Death
10
9
8
7
6
5
4
3
2
1
0
UI
JAL2287
2549
MYC431
D
P.L/Cell Section
30
25
20
15
10
5
0
H37Rv
JAL2261
1934
BND320
BND433
JAL2287
2549
Myc431
E
500 nm
500 nm
500 nm

Figure S3.

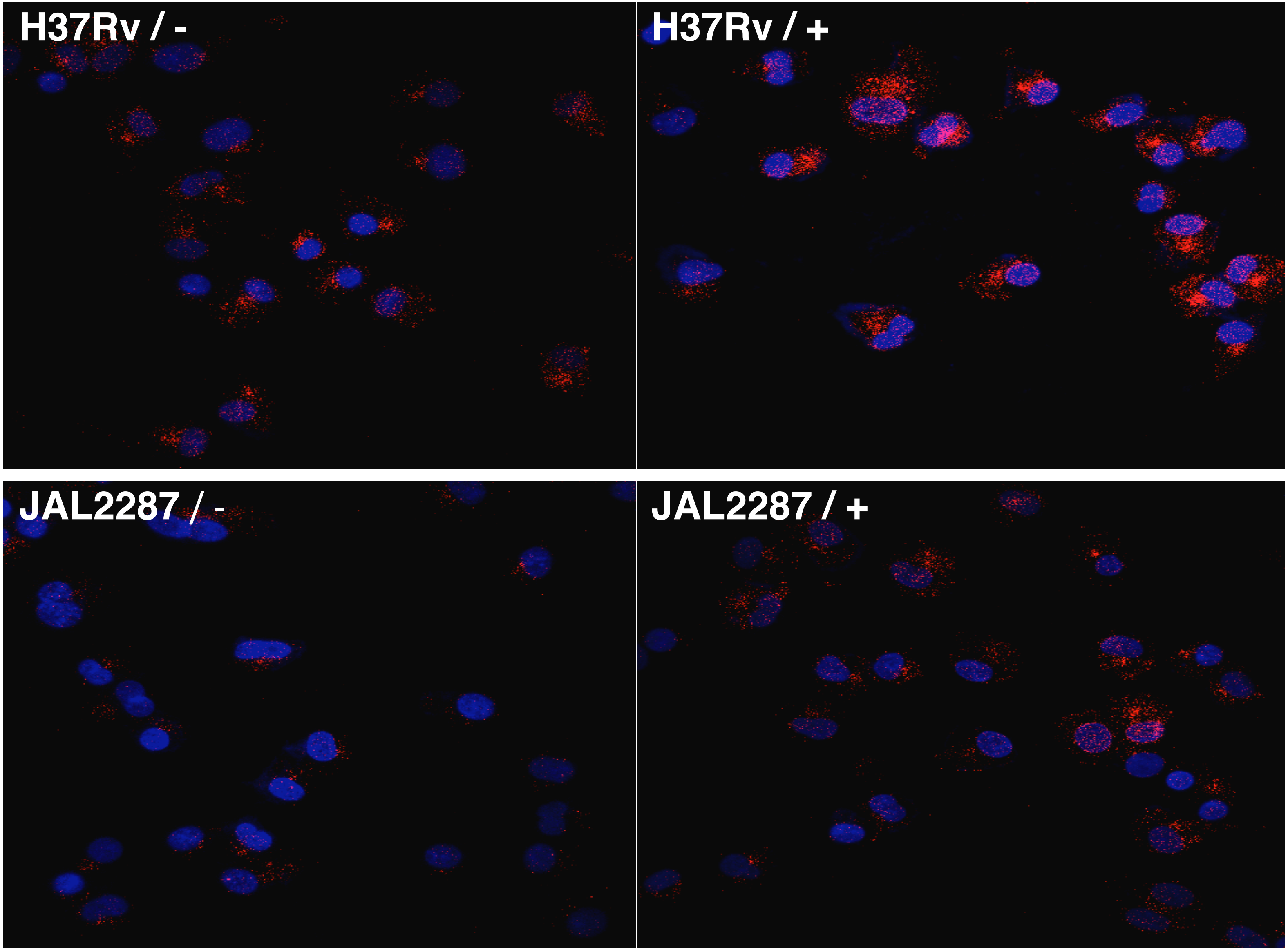

Figure S4.

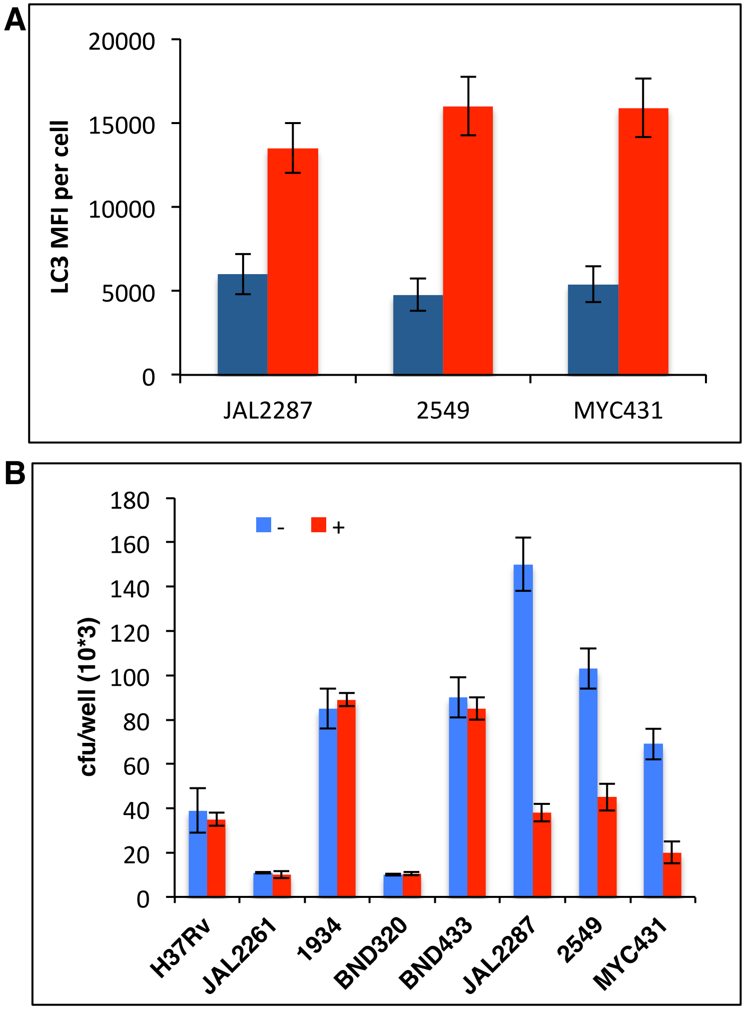
A
LC3 MFI per cell
20000
15000
10000
5000
0
JAL2287
2549
MYC431
B
cfu/well (10*3)
180
160
140
120
100
80
60
40
20
0
-
+
H37Rv
JAL2261
1934
BND320
BND433
JAL2287
2549
MYC431
